# How Women Rest

**Published:** 1889-05

**Authors:** 


### HOW WOMEN REST.

How differently men and women indulge themselves in what is called a resting spell. "I guess I'll sit down and mend these stockings and rest awhile," says the wife ; but her husband throws himself upon the easy lounge, or sits back in his armchair, with hands at rest and feet placed horizontally upon another chair. The result is that his whole body gains full benefit of the half-hour he allows himself from work, and the wife only receives that indirect help which comes from change of occupation. A physican would tell her that taking even ten minutes' rest in a horizontal position, as a change from standing or sitting at work, would prove more beneficial to her than any of her makeshifts at resting. Busy women have a habit of keeping on their feet just as long as they can, in spite of backaches and warning pains. As they grow older they see the folly of permitting such drafts upon their strength, and learn to take things easier, let what will happen. They say, "I used to think I must do thus and so, but I've grown wiser and learned to slight things." The first years of housekeeping are truly the hardest, for untried and unfamiliar cares are almost daily thrust upon the mother and homemaker—*New York Graphic.*

